# Thioredoxin 1 (TRX1) Overexpression Cancels the Slow Force Response (SFR) Development

**DOI:** 10.3389/fcvm.2021.622583

**Published:** 2021-02-26

**Authors:** Maite R. Zavala, Romina G. Díaz, María C. Villa-Abrille, Néstor G. Pérez

**Affiliations:** ^1^Fellow From Consejo Nacional de Investigaciones Científicas y Técnicas (CONICET), Buenos Aires, Argentina; ^2^Established Investigators of CONICET, Buenos Aires, Argentina; ^3^Full Professors of Physiology, Facultad de Ciencias Médicas de La Plata, Universidad Nacional de La Plata, Buenos Aires, Argentina

**Keywords:** TRX1, SFR, NHE1, antioxidant, cardiac hypertrophy

## Abstract

The stretch of cardiac muscle increases developed force in two phases. The first phase occurs immediately after stretch and is the expression of the Frank–Starling mechanism, while the second one or slow force response (SFR) occurs gradually and is due to an increase in the calcium transient amplitude. An important step in the chain of events leading to the SFR generation is the increased production of reactive oxygen species (ROS) leading to redox sensitive ERK1/2, p90RSK, and NHE1 phosphorylation/activation. Conversely, suppression of ROS production blunts the SFR. The purpose of this study was to explore whether overexpression of the ubiquitously expressed antioxidant molecule thioredoxin-1 (TRX1) affects the SFR development and NHE1 phosphorylation. We did not detect any change in basal phopho-ERK1/2, phopho-p90RSK, and NHE1 expression in mice with TRX1 overexpression compared to wild type (WT). Isolated papillary muscles from WT or TRX1-overexpressing mice were stretched from 92 to 98% of its maximal length. A prominent SFR was observed in WT mice that was completely canceled in TRX1 animals. Interestingly, myocardial stretch induced a significant increase in NHE1 phosphorylation in WT mice that was not detected in TRX1-overexpressing mice. These novel results suggest that magnification of cardiac antioxidant defense power by overexpression of TRX1 precludes NHE1 phosphorylation/activation after stretch, consequently blunting the SFR development.

## Introduction

The slow force response (SFR) to myocardial stretch is a second increase in developed force that occurs just after the Frank–Starling mechanism takes place. It is well-known that an augmented calcium transient amplitude underlies its development (1, 2) but the exact genesis of this increase is still a matter of debate. We have proposed that the SFR is the mechanical expression of a stretch-triggered autocrine mechanism where oxidative stress targeting the Na^+^/H^+^ exchanger (NHE1) plays a crucial role (3). We demonstrated that an increased production of reactive oxygen species (ROS) is critical in the chain of events leading to SFR generation (4, 5). Conversely, suppression of ROS production blunted the SFR as shown by us (4) and others (6). We also provided evidence about mitochondrial origin of ROS but induced by a small amount of NADPH oxidase-derived ROS (4), which clearly resembles the so-called “ROS-induced ROS-release” phenomenon (7–9).

On the other hand, cardiac hypertrophy and failure (one of the most important health problems in the world) are both triggered by intracellular signals that occur after myocardial stretch. Moreover, increased oxidative stress has been reported to play a critical role in the development of heart disease (10–13). Therefore, like a double-edged sword, mechanical stress may not only trigger immediate intrinsic heart mechanisms to adapt cardiac output to changes in hemodynamic conditions, but also would constitute the foundation stone toward dysfunction if the initial events are perpetuated in time. In this scenario, a precise characterization of the stretch-triggered signaling pathway may serve to better understand cardiac physiology, but also would pave the way to develop innovative strategies to prevent cardiac pathophysiology.

Thioredoxin-1 (TRX1) is one of the most important cellular antioxidant molecules (14). This ubiquitously expressed antioxidant enzyme is responsible for maintaining the intracellular compartment in a reduced state (14, 15). Interestingly, it has been shown that overexpression of this protein negatively regulates cardiac hypertrophy (10, 12). For the present study, we hypothesized that exacerbation of cardiac antioxidant power by overexpressing TRX1 in mice would cancel the SFR to stretch. We will provide evidence that it was indeed the case.

## Methods

All experiments were performed following the *Guide for the Care and Use of Laboratory Animals* (National Research Council, 2011, Eighth Edition. Washington, DC: The National Academies Press) (16). The experimental protocols was approved by the Animal Welfare Committee of La Plata School of Medicine.

### Animals

Four-month-old male transgenic mice overexpressing Thioredoxin 1 (TRX1) and wild-type (WT) controls were generously provided by “Institute of Cardiovascular Physiopathology,” University of Buenos Aires. These transgenic mice were originally generated in Dr. Sadoshima's Laboratory on an FVB background using the α-myosin heavy-chain promoter to achieve cardiac-specific expression (10). Animals of ~25–30 g of body weight (*n* = 15) were sacrificed by cervical dislocation. The chests were opened to excise the heart, and the papillary muscles were removed carefully from the left ventricle. In addition, thin tissue slices of left ventricle were cut, frozen, and stored at −80°C for later immunoblotting measurements.

### Echocardiography and Postmortem Cardiac Hypertrophy Indexes Determinations

Transthoracic echocardiography was performed immediately before sacrifice, under light anesthesia with 287.5 mg/kg of 2.5% filtered 2,2,2-tribromoethano. As described previously (11), two-dimensional parasternal short-axis imaging plane was used to obtain M-mode tracings at the level of the papillary muscles, with 14-MHz linear transducer. Measurements were performed according to the American Society of Echocardiography method (17). The following parameters were measured: left ventricular end diastolic diameter (LVEDD), left ventricular end systolic diameter (LVESD), left ventricular end diastolic wall thickness (LVEDW), and left ventricular end systolic wall thickness (LVESW). Heart rate (HR), fractional shortening (FS), and ejection fraction (EF) were also determined. After sacrifice, body weight (BW), heart weight (HW), tibia length (TL), and left ventricular weight (LVW) were measured to calculate LVW/BW and LVW/TL ratios.

### SFR Development

Papillary muscles from the left ventricle were used to assess the SFR to stretch as previously described (18). Briefly, the muscles were mounted in a thermostatic perfusion chamber attached to a force transducer, and perfused (at a constant rate of 5 ml/min) with Ringer's buffered solution containing (mmol/L): NaCl 128.30, KCl 4.50, CaCl_2_ 1.35, NaHCO_3_ 20.23, MgSO_4_ 1.05, and glucose 11.00 and equilibrated with 5% CO_2_-95% O_2_ (pH ~7.40, 30°). To avoid the possible participation of catecholamines, we used 1 μmol/L prazosin and 1 μmol/L atenolol. The muscles were stimulated at 0.2 Hz (with a voltage 10% over the threshold) and isometric contractions were measured. Force records obtained with a silicon strain gauge (model AEM 801, Kronex Technologies Corp, Oakland, CA, USA) were normalized by the cross-sectional area, which was calculated as 0.75 of the products of thickness and width (in mm^2^): WT mice 0.17 ± 0.02, *n* = 7; TRX1-overexpressing mice 0.22 ± 0.02, *n* = 8, *p* = 0.11.

After mounting, the slack length of each muscle was determined and then the muscles were progressively stretched to the length at which they developed maximal twitch force (*L*_max_). After a few minutes at this length, the muscles were shortened to 95% of their maximal twitch force (length that approximated 98% of *L*_max_ and referred to as L98). Then, they were shortened to 92% of *L*_max_ (L92) and maintained at this length until the beginning of the experimental protocol to determine the SFR, when they were abruptly stretched from L92 to L98 for 10 min. After the stretching protocol, the papillary muscles were frozen in liquid nitrogen and stored at −80°C for later immunoblotting measurements.

### Western Blotting and Immunoprecipitation Assays

Papillary muscles (previously subjected to the stretching protocol) and ventricular slices were homogenized in lysis buffer solution containing (mmol/L): Tris–HCl 50.0, EGTA 5.0, EDTA 2.0, NaF 100, Na_3_VO_4_ 1, and Triton X-100 0.05% (pH = 7.5) or in RIPA buffer (Santa Cruz Biotechnology, sc-24948), respectively. Both solutions were supplemented with protease inhibitor cocktail, PMSF, and sodium orthovanadate. After cold centrifugation, the supernatant was collected and used to determine protein concentration by Bradford's method (Bio-Rad dye reagent) as described by the manufacturer, using BSA as a standard. To determine NHE1 phosphorylation after stretch, the homogenate of papillary muscles containing 300 μg of total protein was immunoprecipitated using the NHE1 polyclonal antibody as previously described (5, 18). Because protein amount from one stretched papillary muscle is not enough to do the immunoprecipitation, two muscles from different mice (but belonging to the same group) were homogenized together for each sample. This intervention considerably reduced the final size of samples. Immunoprecipitated samples, as well as homogenate tissue slices, were denatured, and equal amounts of protein were subjected to SDS-PAGE (8% SDS polyacrylamide gel), and electrotransferred to PVDF membranes. Then, the membranes were blocked with non-fat dry milk (5% in T-TBS) and incubated overnight with specific antibodies against: phospho-14-3-3 binding motif (p-14.3.3 BM, Cell Signaling, #9601), phospho-ERK1 and ERK2 (p-ERK1/2, Santa Cruz Biotechnology, sc-16982), phospho-p90RSK (p-p90RSK, Cell Signaling #9341), and NHE1 (NHE1, Santa Cruz Biotechnology, sc-28758). To normalize the signal of different proteins, Actin (Abcam, ab170325) or NHE1 as a loading control were assayed, respectively.

Peroxidase-conjugated anti-rabbit (NA934, GE Healthcare Life Sciences) or anti-mouse (NA931, GE Healthcare Life Sciences) was used as secondary antibodies. Bands were visualized using the Immobilon Western chemiluminescent HRP substrate (Millipore, WBKLS0100). Autoradiograms were analyzed by densitometry (Scion Image).

### Statistics

Data are expressed as mean ± SEM. Student's *t* test was used to compare unpaired samples between two groups. Non-parametric Mann–Whitney test was used to compare data with *n* < 4. A *p* < 0.05 was used to consider statistically significant.

## Results

Echocardiographic parameters determined just before sacrifice and postmortem index determinations are shown in Table 1. Similar LVEDD, LVESD, and LVESW were detected in both experimental groups. A slight but significant increase in the LVEDW was detected in TRX1-overexpressing mice, but postmortem determination of cardiac hypertrophy indexes (LVW/BW and LVW/TL) did not show any difference between groups. Furthermore, similar cardiac function was observed in both animal groups (Table 1).

**Table 1:** Echocardiographic and postmortem cardiac parameters.

	**WT (*n* = 5)**	**TRX1 (*n* = 5)**
LVEDD (mm)	3.50 ± 0.15	3.58 ± 0.15
LVESD (mm)	2.16 ± 0.17	2.37 ± 0.13
LVEDW (mm)	0.93 ± 0.03	1.07 ± 0.05 (*p* = 0.047)
LVESW (mm)	1.24 ± 0.05	1.28 ± 0.04
HR (beats per min)	482.40 ± 33.75	442.40 ± 21.41
EF%	76.45 ± 2.73	70.93 ± 1.88
%FS	38.62 ± 2.40	33.89 ± 1.49
	**WT (*****n*** **=** **7)**	**TRX1 (*****n*** **=** **8)**
BW (g)	28.03 ± 0.93	26.30 ± 1.15
TL (mm) HW (mg) LVW (mg) LVM/BW (mg/g)	20.20 ± 0.63 128.57 ± 3.40 85.71 ± 2.97 3.06 ± 0.09	20.23 ± 0.57 125.00 ± 7.56 85.00 ± 5.00 3.26 ± 0.20
LVM/TL (mg/mm)	4.27 ± 0.20	4.20 ± 0.23

*Male 4-month-old mice were used. LVEDD, Left ventricular end diastolic diameter; LVESD, Left ventricular end systolic diameter; LVEDW, Left ventricular end diastolic wall thickness; LVESW, Left ventricular end systolic wall thickness; HR, Heart rate; EF, Ejection fraction; FS: fractional shortening. BW, Body weight; TL, Tibia length; HW, Heart weight; LVW, Left ventricular weight. Data are expressed as means ± SEM*.

**Figure 1:**
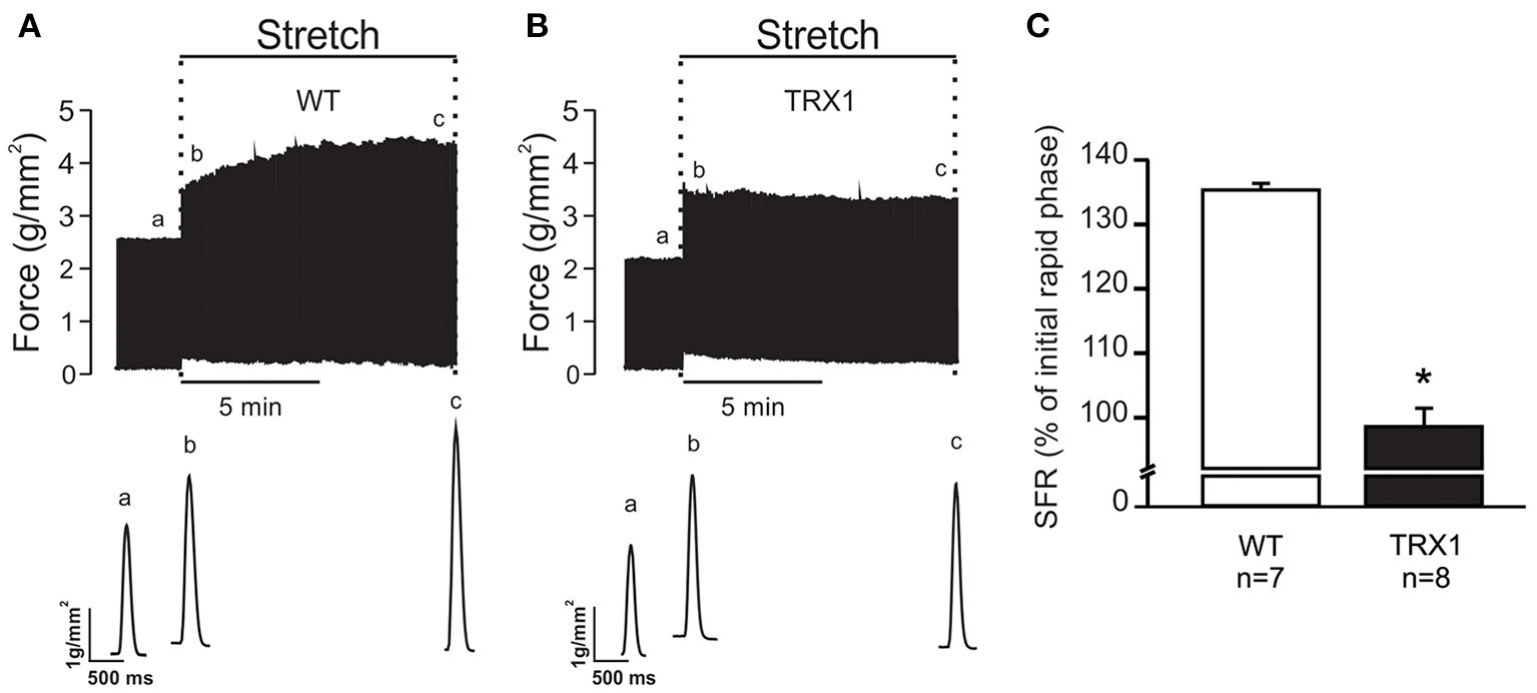
SFR development of isolated papillary muscles from WT and TRX1-overexpressing mice. **(A)** Original force record of an isolated papillary muscle from a WT mouse that was suddenly stretched from 92 to 98% of *L*_max_. The characteristic two-phase increase in force that follows myocardial stretch can be observed. Individual traces corresponding to developed force indicated in the upper panel with “a” (before stretch), “b” (immediately after stretch), and “c” (10 min after stretch) are shown in the bottom panel. **(B)** Same as A, but in a papillary muscle from a TRX1-overexpressing mouse where the SFR development was completely canceled. **(C)** Averaged SFR results after 10 min of stretch from both experimental groups, expressed as percent of the initial rapid phase. *Indicates *p* < 0.05 vs. WT.

The next step of the study was to verify whether overexpression of TRX1 affects the development of the SFR to myocardial stretch. Therefore, isolated papillary muscles from WT and TRX1-overexpressing mice were stretched from 92 to 98% of *L*_max_. Figure 1A shows an original force record of a muscle from a WT mouse where the initial rapid increase in force (expression of the Frank–Starling mechanism) can be appreciated followed by the SFR that stabilized after ~10 min. Interestingly, when the same protocol was performed in muscles from TRX1-overexpressing hearts, a similar initial increase in force was detected, but the SFR was completely canceled (Figure 1B). Non-significant changes in time to half relaxation were observed during the SFR in WT or TRX1-overexpressing mice (in msec, measured immediately and after 10 min of stretch): from 103.6 ± 27.8 to 107.1 ± 33.1, *n* = 7 in WT; from 97.5 ± 23.7 to 98.8 ± 26.2, *n* = 8 in TRX1. These findings indirectly disregard the possible participation of changes in myofilament calcium responsiveness in the observed effects of stretch on force development.

**Figure 2:**
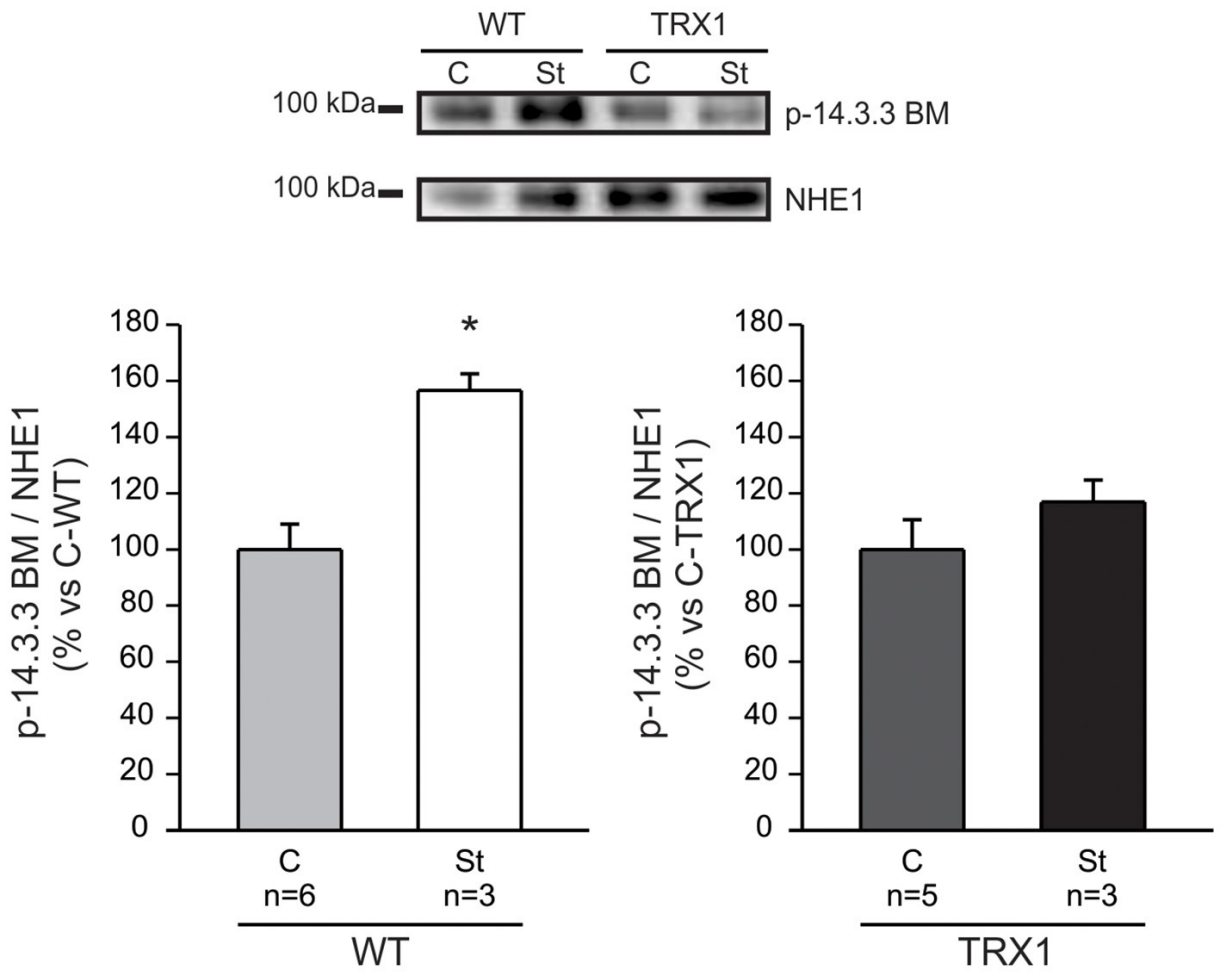
NHE1 phosphorylation after stretch. Myocardial stretch promoted a significant increase in NHE1 phosphorylation in WT mice (“St-WT”) compared to non-stretched controls (“C-WT”), as shown in the original immunoblot (upper panel) and in the averaged results of the lower left panel. Interestingly, the stretch of muscles overexpressing TRX1 (“St-TRX1”) did not show significant changes in NHE1 phosphorylation compared to its corresponding non-stretched controls (“C-TRX1”), as shown in the immunoblot (upper panel), and in the averaged results (lower right panel). *Indicates *p* < 0.05 vs. C-WT.

Previous reports assigned a crucial role to NHE1 phosphorylation/activation at Ser703 in the chain of events leading to the SFR development (5). Therefore, we sought to quantify NHE1 phosphorylation at this site in our experimental conditions. As expected, a significant increase in Ser703 NHE1 phosphorylation was detected after stretch in papillary muscles from WT mice compared to non-stretched controls (Figure 2). Remarkably, this effect was not observed in muscles overexpressing TRX1 (Figure 2), which would conceivably justify the observed lack of SFR development in this experimental group.

**Figure 3:**
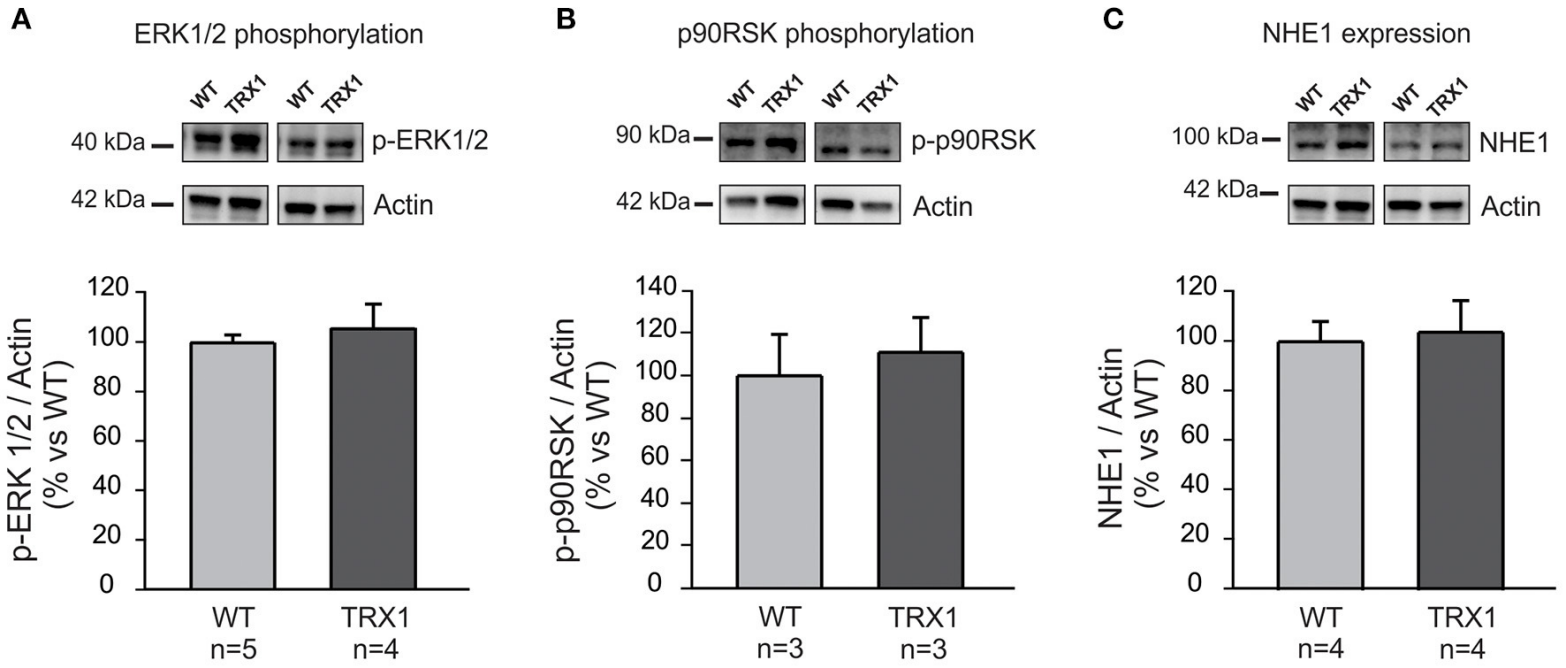
ERK1/2, p90RSK phosphorylation, and NHE1 expression. Basal ERK1/2 **(A)** and p90RSK **(B)** phosphorylation, as well as NHE1 expression **(C)** were similar in both experimental groups. Original blots (top) and averaged data (bottom).

The question as to why TRX1 overexpression prevented NHE1 phosphorylation after stretch was certainly raised. Phosphorylation of the redox-sensitive kinases ERK1/2-p90RSK is a pre-requisite to increase Ser703 exchanger phosphorylation/activation following myocardial stretch (4, 5). Therefore, we decided to prove whether TRX1 overexpression modified basal ERK1/2 or p90RSK phosphorylation/activation, but it was not the case as shown in Figures 3A,B.

Another possibility to explain the lack of increase in NHE1 phosphorylation after stretching could be a decrease in basal NHE1 expression in TRX1-overexpressing animals. However, basal NHE1 expression was similar in both experimental groups as shown in Figure 3C.

Taken together, our results suggest that potentiation of the antioxidant power of the heart by overexpressing TRX1 negatively regulates the SFR development. The underlying mechanism would primarily be assigned to a redox-linked prevention of NHE1 phosphorylation/activation after stretch.

## Discussion

Current findings provide evidence that specific overexpression of cardiac TRX1 blunts the SFR to myocardial stretch by preventing NHE1 phosphorylation/activation.

The SFR constitutes a powerful physiological mechanism by which the heart adapts to an abrupt increase in afterload, which works in concert with the well-known Frank–Starling mechanism. Our interest in deciphering the subcellular basis of this important mechanism is beyond its physiological role, given that crucial signals leading to the SFR (i.e., oxidative stress, NHE1 hyperactivity, augmented Ca^2+^ concentration) play critical roles in the development of pathological cardiac hypertrophy (19, 20). Although speculative, the long journey toward cardiac hypertrophy and failure begins with one step, which may well be the autocrine intracellular signaling pathway triggered by myocardial stretch (3).

TRX1 is an enzyme from the “TRX/TRX reductase” system that together with the “glutathione/glutathione reductase” system represents most of the antioxidant power of cells (21–24). This small cytosolic protein whose main function is to maintain the intracellular medium in a reduced state plays a critical protective role against oxidative stress, which constitutes the primary stimulus to inducing enzyme transcription. The TRXs have captured the interest of the scientific community due to its potentiality as a therapeutic target in clinical medicine (25). In cardiac tissue, TRX1 not only functions as a major antioxidant enzyme but also interacts with important signaling molecules and transcription factors, thereby modulating various cellular functions (14). As stated before, in a previous study, we demonstrated the crucial role played by ROS in the signaling pathway leading to the SFR in the rat myocardium (4). Using pharmacological tools, we were able to demonstrate that prevention of ROS generation after stretch blunts the stretch-induced NHE1 activation and hence the intracellular sodium increase that underlies the SFR development. This effect was primarily assigned to a decrease in the phosphorylation state of the redox-sensitive kinases ERK1/2-p90RSK upstream NHE1. In the present study, we detected that the SFR to stretch was accompanied by an increase in NHE1 phosphorylation in WT mice that was absent in mice overexpressing TRX1 (Figure 2) and that this effect was accompanied by complete suppression of the SFR (Figure 1). It may be argued that chronic exacerbation of the antioxidant defense could induce changes in certain mediators of the SFR. In this regard, the absence of increase in NHE1 phosphorylation after stretch could conceivably be a consequence of a modification of either NHE1 or its upstream kinases ERK1/2-p90RSK. Interestingly, basal ERK1/2-p90RSK phosphorylation was unaltered by TRX1 overexpression as shown in Figure 3. This suggests that overactivation of TRX1 prevented ROS formation after stretch, therefore preventing a possible phosphorylation/activation of ERK1/2-p90RSK that precluded NHE1 phosphorylation/activation. Unfortunately, due to methodological inconveniences, we could not measure ERK1/2-p90RSK phosphorylation after stretching.

Yamamoto et al. (10) have demonstrated many years ago that mice overexpressing TRX1 preserve normal structure and function of the heart. We detected a significant—close to the boundary—increase in the LVEDW in TRX1 mice (*p* = 0.047). However, all other parameters indicate the absence of structural and functional alterations under basal conditions in this animal group compared to control. We do not have a clear explanation for this unexpected finding.

The main contribution of the present study is to demonstrate that exacerbation of myocardial antioxidant defense precludes stretch-induced NHE1 phosphorylation/activation and, consequently, the SFR development. These findings further support the notion that ROS generation after stretch is crucial to adapt cardiac force to changes in hemodynamic conditions.

From a clinical perspective, the fact that severe cardiac disorders like hypertrophy and failure share a common signaling pathway with the SFR would suggest that modulation of TRX1 activity would be a suitable target to develop novel therapeutic strategies against these diseases.

### Limitations of the Study

We did not measure ROS production in our experimental conditions, which may be interpreted as a weakness of the study. However, Perez et al. (26) demonstrated that mice overexpressing TRX1 exhibit similar enzyme activity to WT animals under basal conditions. Interestingly, they also observed a correlation between ROS production and TRX1 activity in a setting of ischemia–reperfusion injury, suggesting that the model needs a stimulus to be switched on. In our experimental conditions, the stretch-triggered ROS production seems to be the initiator of the signaling pathway leading to the SFR. It is important to highlight that if ROS production is prevented either through pharmacological strategies (4) or by overexpressing TRX1 (this study), the SFR is abolished.

We did not explore the role of other important mechanisms of cardiac function such as mitochondrial biogenesis, ATP production, or apoptosis (10, 27, 28), all of which are modulated by TRX1 activity. Further research is necessary to unveil this unresolved question.

## Data Availability Statement

The raw data supporting the conclusions of this article will be made available by the authors, without undue reservation.

## Ethics Statement

The animal study was reviewed and approved by Animal Welfare Committee of La Plata School of Medicine.

## Author's Note

The content of this manuscript has been presented in part at the ISHR World Congress Annual Meeting, April 18–21, 2016, Buenos Aires, Argentina; JMCC 98(2016), S1-S85, WE-072.

## Author Contributions

MRZ performed experiments, analyzed data and wrote de manuscript. RGD performed experiments and analyzed data. MCV-A and NGP designed the experiments, analyzed results and wrote the manuscript.

## Conflict of Interest

The authors declare that the research was conducted in the absence of any commercial or financial relationships that could be construed as a potential conflict of interest.
